# A Human Neural Tube Model Using 4D Self‐Folding Smart Scaffolds

**DOI:** 10.1002/adhm.202501405

**Published:** 2025-10-23

**Authors:** Claudia Dell'Amico, Irene Chiesa, Angela Toffano, Alessio Esposito, Piera Mancini, Chiara Magliaro, Angeliki Louvi, Carmelo De Maria, Marco Onorati

**Affiliations:** ^1^ University of Pisa Department of Biology, Unit of Cell, Molecular, and Developmental Biology Pisa 56127 Italy; ^2^ University of Pisa Department of Clinical and Experimental Medicine Pisa 56126 Italy; ^3^ University of Pisa Department of Information Engineering and Research Center Enrico Piaggio Pisa 56122 Italy; ^4^ Departments of Neurosurgery and of Neuroscience Yale School of Medicine New Haven Connecticut 06520‐8082 United States

**Keywords:** 4D scaffold, bioprinting, iPSCs, neurodevelopment, self‐folding, WDR62

## Abstract

The human brain originates from the neural tube that detaches from the ectodermal layer and gradually develops into a mature structure through highly regulated molecular and cellular processes. Here, stem cell technology is combined with 4D bioprinting, a fabrication process that utilizes additive manufacturing, to generate a 4D‐neural tube (4D‐NT). This consists of a scaffold that can self‐fold over time, which is then populated with iPSC‐derived neuroprogenitors, mimicking neural tube cellular architecture. The scaffold's “smart” self‐folding behavior is driven by the differential swelling properties of bilayer films, which create a deformation gradient upon hydration. Cellular analyses reveal a highly efficient induction of neuroprogenitors on 4D‐NTs, demonstrating the ability of this model to mimic the spatial and structural complexity of the developing human neural tube. Furthermore, 4D‐NTs seeded with iPSCs with a mutation in *WDR62*, associated with autosomal recessive primary microcephaly (MCPH), recapitulate the earlier observations obtained in 2D/3D neural cultures, thereby validating the newly developed 4D‐NT platform and suggesting it represents a tool that can facilitate understanding of human neural development and disease.

## Graphical abstract

Induced pluripotent stem cells (iPSCs) exhibit features comparable to the inner cell mass of the human embryo. iPSCs are applied to a novel self‐folding 4D‐Neural Tube (4D‐NT) structure that mimics the neurulation process. This 4D‐NT model recapitulates early events of human neural development and represents a platform to explore neurodevelopmental disorders.

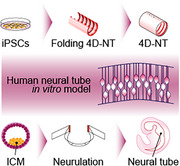

## 1 Introduction

The neural tube serves as the primordium from which the brain and spinal cord develop to form the central nervous system (CNS) during human prenatal development.

Once the three embryonic germ layers are established, the dorsal ectoderm gives rise to the neural plate. Subsequently, the neural plate folds to form the neural tube during primary neurulation, around the third post‐conceptional week (pcw) in humans.^[^ ^1^, ^2^ ^]^ Neural tube formation is a crucial step in neurogenesis, regulated by various molecular and cellular mechanisms that coordinate proper neural plate closure and regionalization along both the dorso‐ventral and antero‐posterior axes.^[^ ^3^ ^]^ As development progresses, the neural tube gives rise to the encephalic vesicles—forebrain, midbrain, and hindbrain—as well as the spinal cord.^[^ ^4^ ^]^

The neural tube is initially composed of neuroepithelial cells – multipotent neural progenitor cells (NPCs) – that undergo mostly symmetric divisions to expand the NPC pool, as well as asymmetric divisions to generate the first neuroblasts. Later, neuroepithelial cells transition into radial glial cells that give rise to neurons and macroglia (i.e., astrocytes and oligodendrocytes) and also provide scaffolding for migrating nascent neurons. These NPC populations form a polarized structure and exhibit an elongated bipolar morphology: one process is directed toward the lumen of the neural tube (ventricular ridge) and the other toward the external (pial) surface. NPCs maintain apico‐basal polarity, with the apical side facing the neural tube lumen, and express key markers such as nestin (an intermediate filament protein), along with transcription factors like SOX2 (SRY‐box 2).^[^ ^5^, ^6^ ^]^

Genetic or environmental insults leading to cellular or molecular alterations in NPCs can disrupt neurodevelopment, giving rise to a spectrum of abnormalities spanning from lethal ones, such as anencephaly and craniorachischisis, to a broad array of morphologically distinct malformations.^[^ ^3^, ^7^ ^]^ Animal models have been widely employed to investigate the molecular mechanisms underlying neural tube pathologies, allowing the identification of several candidate genes playing roles in adhesion, cilia formation, cell polarization, and many other processes supporting the proper formation of the neural tube.^[^ ^8^, ^9^, ^10^, ^11^, ^12^, ^13^ ^]^ Nevertheless, the human CNS – particularly the brain – displays unique species‐specific features. Additionally, human neural tissue itself and the cellular populations generated sequentially during brain development are mostly inaccessible for experimental studies.

Recent advances in human induced pluripotent stem cell (iPSC) technology have enabled the generation of in vitro models for optimal characterization of human‐specific aspects of neural tube development and related pathologies. Specifically, 2D (i.e., monolayer), as well as 3D (i.e., brain organoids) neural systems have been extensively employed. More recently, bioengineered 3D systems have been developed. Their cost, with respect to canonical 2D and 3D cultures, however, remains high, despite their potential.^[^ ^14^, ^15^ ^]^

In this study, we sought to develop a dynamic model of early human developing neural tube, taking advantage of iPSCs combined with a bioengineered multi‐material scaffold able to self‐fold, aimed at recreating tissue architecture during neural tube closure. The design of this dynamic model arises from a well‐established concept regarding bilayer films.^[^ ^16^, ^17^ ^]^ When hydrated, a bilayer film composed of materials with different swelling behavior undergoes folding and rolling movements caused by the deformation mismatch in its layers. Here, to fabricate such a bilayer film, we exploited a novel fabrication approach, referred to as 4D bioprinting, in which additive manufacturing technologies are used to fabricate active scaffolds, designed to interact with physiological cellular systems, changing their properties and, macroscopically, their shape over time (i.e., the fourth dimension) depending on external stimuli.^[^ ^18^, ^19^ ^]^

This model also enables the identification of key players in potentially compromised pathways, highlighting them as valuable targets for studying rare genetic neurodevelopmental disorders such as primary microcephaly (MCPH). Affected individuals present with reduced head circumference and brain size, perturbations in neuronal cell number, and abnormal neocortical cytoarchitecture.^[^ ^20^, ^21^ ^]^ The second most common form of MCPH is linked to recessive mutations in *WDR62*,^[^ ^22^ ^]^ encoding a spindle pole‐associated scaffold protein with pleiotropic functions, ranging from multiprotein complex assembly at the mitotic spindle poles, biogenesis of primary cilia, and choice of proliferative versus differentiative progenitor cell division.^[^ ^23^, ^24^, ^25^, ^26^, ^27^, ^28^ ^]^ Here, we employed the 4D‐NT platform to investigate early cellular and molecular events underlying WDR62‐associated MCPH, as a proof of concept for the applicability of this system to investigate neurodevelopmental pathologies.

## 2 Results

### 2.1 Generation of Human 4D‐NTs

**Figure 1:**
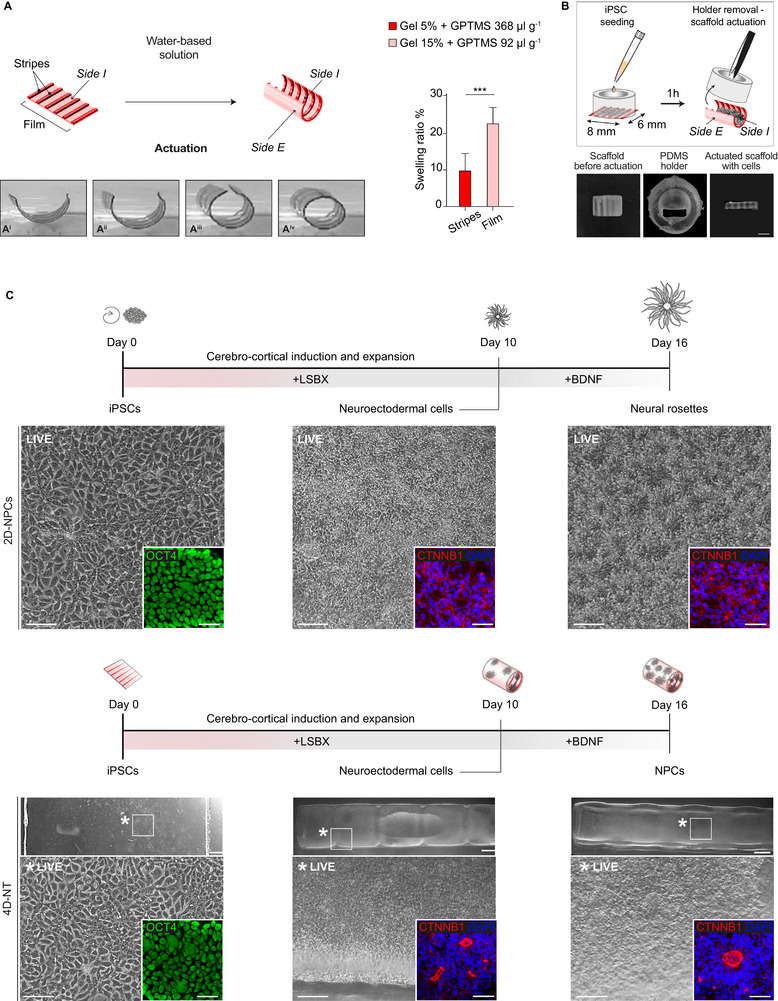
Establishment of the 4D‐NT system as a model of neural tube development. A) Schematic and representative frames (A^i^: 2s; A^ii^: 4s; A^iii^: 6s; A^iv^: 8s) depicting the general scaffold structure and folding process occurring after hydration. The presence of lower‐swelling stripes (Gel 5% + 3‐glycidoxypropyl)trimethoxysilane – GPTMS – 368 µl g^−1^) (*Side I*) allows control over the folding direction of the scaffold. B) Schematic representing the seeding process of iPSCs on the internal surface of the scaffold (*Side I*). Given the intrinsic property of the scaffold when hydrated (actuation), a holder is used to keep the scaffold flat during cell plating. 1 h after seeding, the holder is removed, and the scaffold is allowed to actuate. C) Schematic and representative bright‐field (LIVE) and confocal immunofluorescence (OCT4 and CTNNB1) images of CTRL_1_ iPSC differentiation in 2D‐NPC and 4D‐NT conditions. After plating, iPSCs are driven to neural fate via a Dual SMAD inhibition‐based protocol (LSBX, for LDN193189, SB431542, XAV939). After 10 days, cells undergo morphological changes, acquiring neuroectodermal identity, showing neural rosette formation (CTNNB1 staining). From Day 10 onward, neuroectodermal cells are exposed to BDNF and differentiate into neural progenitor cells (NPCs) organized in polarized rosettes, thus establishing the 4D‐NT final structure. Scale bar: 1 mm in A, 1.5 mm in B, 50 µm in C, and 10 µm in insets.

To develop the 4D‐NT system, we undertook 4D bioprinting to fabricate a self‐folding scaffold based on natural polymers. As described above, the *smart* behavior is achieved by the differential swelling properties of bilayer films. Thus, when the scaffold is dipped into a water‐based solution, the swelling mismatch creates a deformation gradient in the structure that causes the film to fold/roll (**Figure** **1**A; Video S1, Supporting Information). To generate the 4D‐NT, the two layers of the scaffold were made of the same bulk material (gelatin cross‐linked using GPTMS), thus ensuring a chemical bond between the layers and avoiding delamination. The difference in swelling was achieved by adjusting the concentrations of gelatin and GPTMS. The first layer (Side E, Gel 15% + GPTMS 92 µl g^−1^) was generated as a continuous film, while the second layer (Side I, Gel 5% + GPTMS 368 µl g^−1^) was bioprinted on top as stripes. The orientation of these stripes was used to control the folding direction (parallel to the stripes), which in turn defined the internal side (Side I) and external side (Side E) (Figure 1A). Additionally, when the stripes were printed parallel to the shorter side of the scaffold, the resulting structure exhibited a smaller radius of curvature, ≈1.5 mm.

For iPSC seeding, a customized holder (see Supporting Information for the fabrication) was applied on top of the scaffold to temporarily prevent folding upon hydration. Then, undifferentiated iPSCs (CTRL_1_ iPSCs) were plated as a confluent monolayer on Side I of the scaffold seeding area. After cell adhesion, the scaffolds were actuated by removing the holders (Figure 1B), and the seeded iPSCs on the forming 4D‐NTs were driven to neural fate. On Day 0 of neural induction, a confluent monolayer of undifferentiated iPSCs, positive for octamer‐binding transcription factor 4 (OCT4), a marker of pluripotency, was visible (Figure 1C, bottom left panel). Subsequently, cells underwent morphological changes, acquired a defined apico‐basal polarity, and started to arrange into neural rosettes, as shown by β‐catenin (CTNNB1) immunostaining, indicative of their progressive acquisition of NPC identity. On Day 10, a neuroectodermal layer was established (Figure 1C, mid‐bottom panel). Notably, during the differentiation process, NPCs remained evenly distributed on the surface (Side I) of the folded 4D‐NT. NPCs were then driven toward differentiation by administration of neurotrophins (i.e., BDNF). On Day 16, 4D‐NT samples were further examined for their apico‐basal arrangement through CTNNB1 immunostaining (Figure 1C, bottom right panel).

In parallel, we implemented the well‐established protocol^[^ ^27^, ^29^ ^]^ for neural differentiation in a monolayer, which allowed us to compare the conventional 2D with the newly developed 4D‐NT system. iPSCs were seeded onto Matrigel‐coated multi‐well plates and differentiated toward a neural fate via the same protocol (Figure 1C, top left panel). iPSCs underwent similar morphological changes as those seeded on 4D‐NTs, establishing a confluent NPC monolayer with apico‐basal polarization on Day 10 (hereafter 2D‐NPCs) (Figure 1C, top mid panel) and defined neural rosette formation on Day 16 (Figure 1C, top right panel).

To further characterize the newly developed system, we seeded an additional independent control iPSC line (CTRL_2_ iPSCs) (Figure S1A–C, Supporting Information); we employed a non‐folding gelatin scaffold to generate 2D‐unfolded neural tubes (2D‐uNTs, Figure S1B, Supporting Information), thus generating CTRL_2_ 2D‐uNTs and 4D‐NTs.

From Day 0 to Day 16, CTRL_2_ 2D‐NPCs, 2D‐uNTs, and 4D‐NTs displayed efficient neuralization as shown by extensive presence of neural rosettes, thus indicating that the selected biomaterial (i.e., gelatin cross‐linked with GPTMS) is highly conducive to NPC growth and differentiation.

Collectively, our approach enabled the formation of 4D‐NTs exhibiting a neural tube‐like morphology by Day 16, densely populated with NPCs.

### 2.2 4D‐NTs Resemble Human Embryonic Neural Tube Organization and Efficiently Support Neural Fate Commitment

**Figure 2:**
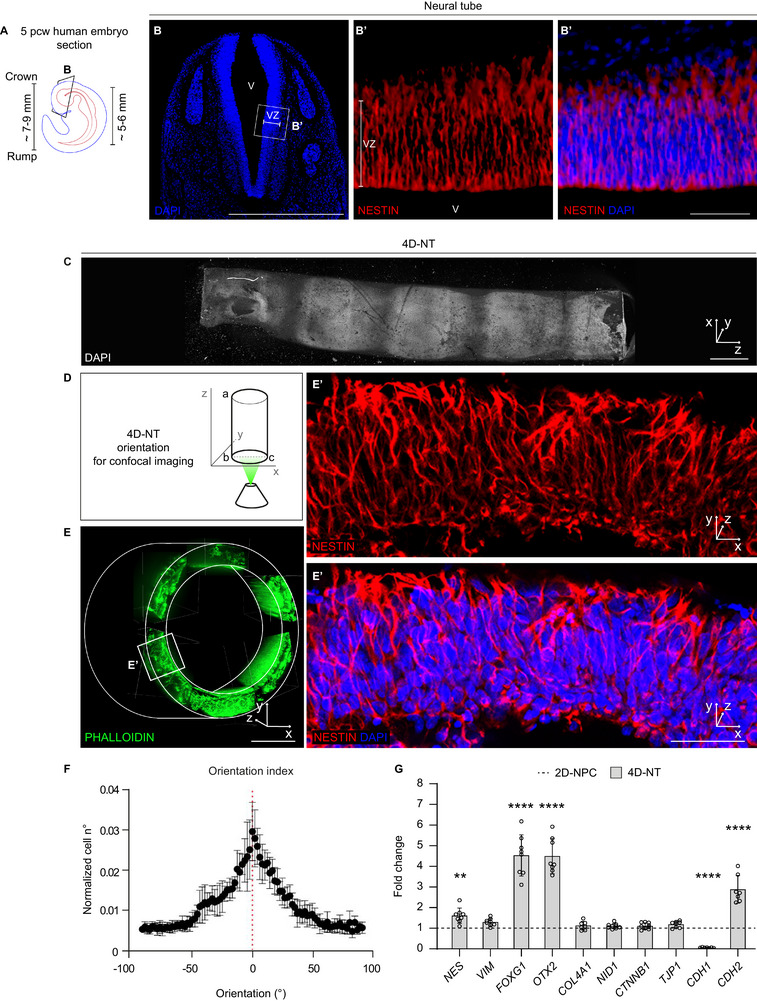
4D‐NTs resemble the human neural tube. A) Schematic representation of the 5 post‐conceptional week (pcw) human embryo. At this stage, crown‐to‐rump length is ≈7–9 mm and neural tube length is ≈5–6 mm. B) Representative immunofluorescence images of the 5 pcw forebrain coronal section highlighting the developing neural tube, the ventricle (V), and the ventricular zone (VZ) constituted by nestin^+^ NPCs displaying a clear radial orientation with respect to the ventricular rim. B’ shows the magnification of the box in B. C) Representative immunofluorescence (DAPI) image of the whole 4D‐NT structure, horizontally oriented. D) Schematic of the orientation of 4D‐NT samples for confocal imaging. E) 3D reconstruction of stitched immunofluorescence images of the whole 4D‐NT structure, stained with phalloidin, oriented with the major axis (a‐b) perpendicular to the *x‐y* plane. In the representative magnified area in E’, nestin^+^ NPCs present an overall radial orientation with respect to the 4D‐NT lumen, mimicking NPC orientation in the developing human neural tube shown in B’. F) Mean orientation index of nestin^+^ NPCs in the 4D‐NT samples obtained by confocal image analysis, showing that cells are preferentially oriented along a specific angle. G) Quantitative analysis of mRNA levels detected by RT‐qPCR of 4D‐NT samples with respect to 2D‐NPCs shows marker gene expression levels on Day 16. Note the epithelial (*CDH1)* to neural (*CDH2*) cadherin switch. Data (mean ± SD, with dots showing the mean of a technical triplicate) are shown as fold change in mRNA expression relative to *GAPDH*, a housekeeping gene, according to the 2^−ΔΔCT^ method. 4D‐NT and 2D‐NPC samples *n* = 6; ^**^ *p*‐value < 0.01, and ^****^ *p*‐value < 0.0001; unpaired t‐test. Scale bars: 500 µm in B, C, and E; 50 µm in B’ and E’. Nuclei are counterstained with DAPI.

During human embryogenesis, the neural tube progressively acquires a folded pattern with spatial tissue organization. We analyzed the human embryonic forebrain at 5 post‐conceptional weeks (pcw, or Carnegie Stage 15) (**Figure** **2**A; Figure S2A–C, Supporting Information), when the human embryo has a crown‐to‐rump length of ≈7–9 mm, with CNS length of ≈6 mm and a diameter of ≈600 µm at the level of the spinal cord^[^ ^35^ ^]^ (Figure 2A; Figure S2A–C, Supporting Information). Around the ventricle (V), NPCs establish the ventricular zone (VZ). At this stage, NPCs (nestin^+^) are organized in a ≈100 µm‐thick pseudostratified neuroepithelium and are radially oriented (Figure 2B, B’; Figure S2A–C, Supporting Information).

CTRL_1_ 4D‐NT samples on Day 10 and on Day 16 were analyzed by whole‐mount immunofluorescence and confocal microscopy to verify their organization and the efficiency in iPSC‐to‐NPC conversion. Horizontally oriented whole‐mount 4D‐NTs showed a homogeneous distribution of nestin^+^ and SOX2^+^ NPCs all along their surface (≈8 mm‐long on the major axis), organized in neural rosettes. NPCs are apico‐basally oriented toward the luminal rim, positive for Tight junction protein 1 (TJP1, also known as zonula occludens‐1, ZO‐1, expressed at the apical domains of the NPCs and demarcating the lumen of the neural tube) and for Pericentrin (PCTN, expressed in the basal body) (Figure S3A–C, Supporting Information).

On Day 16, the non‐folding CTRL_1_ 2D‐uNT exhibited multiple neural rosettes consisting of apico‐basally oriented (CTNBB1^+^) neocortical cells (Forkhead Box G1, FOXG1^+^) across the scaffold surface (Figure S4A–C, Supporting Information). Similarly, CTRL_2_ 2D‐uNT and 4D‐NT samples showed homogeneous distribution of FOXG1^+^, CTNNB1^+^, and TUBB3^+^ cells organized into neural rosettes across the scaffold surface (Figure S5A–E, Supporting Information). Whole‐mount stained 4D‐NTs (Figure 2C) were “vertically” oriented and analyzed through confocal imaging (Figure 2D; Figure S6A,B, Supporting Information). Phalloidin (highlighting F‐actin cytoskeletal components) staining in whole mount spinning disk confocal imaging showed homogeneous distribution of NPCs on the whole 4D‐NT surface (Figure 2E).

As shown in the magnified area in Figure 2E, the thickness of cellularized 4D‐NTs was comparable with the thickness of a 5 pcw neural tissue, i.e., ≈100 µm; nestin^+^ NPCs were also present on the 4D‐NT ridge and appeared radially oriented toward the scaffold lumen, resembling NPC spatial organization in the developing neural tube (Figure 2B and B’). Analysis of the orientation of nestin^+^ processes, indeed, showed that cells did orient perpendicularly to the lumen with a standard error of ± 18° (Figure 2F; Figure S7, Supporting Information).

Next, we explored the properties of the 4D‐NTs by analyzing the expression of markers of neuroepithelium specification and polarity (Figure 2G). During neural tube development, NPCs rely on temporally regulated adhesion mechanisms, underlain by the transition from epithelial cadherin (also known as E‐cadherin, or CDH1) to neural cadherin (also known as N‐cadherin or CDH2).^[^ ^36^ ^]^ Additionally, we used *TJP1* to assess apical polarity. To inspect basal polarity, we analyzed on Day 16 the expression of genes coding for membrane proteins, Collagen type IV alpha 1 chain (*COL4A1*) and Nidogen 1 (*NID1*).^[^ ^37^ ^]^ 4D‐NTs showed comparable or even enhanced NPC gene expression in comparison with 2D‐NPCs (Figure 2G). Furthermore, neuroectodermal gene expression was increased in 4D‐NT with respect to 2D‐NPC samples, as demonstrated by the enhanced expression of *NES, FOXG1*, and *OTX2* (Figure 2G). Of note, in the 4D‐NTs, the levels of *CDH1* were diminished while *CDH2* increased, thus indicating an efficient conversion from epithelial to neural cadherins, as occurs during neural tube development. We additionally evaluated the expression of *CDH2* across 2D‐NPCs, 2D‐uNTs, and 4D‐NTs of CTRL_1_ and CTRL_2_ iPSCs on Day 16, finding similar or increased expression of *CDH2* (Figures S4C and S5E, Supporting Information). Altogether, these data indicate: i) **a comparable, or enhanced, neural conversion** in both 2D‐uNTs and 4D‐NTs compared to 2D‐NPCs, suggesting that the gelatin‐GPTMS scaffolds are conducive for neural differentiation; ii) the generation of 4D‐NTs with **a neural tube‐like identity and tissue architecture** that more closely resembles the native neural tube.

### 2.3 Use of 4D‐NTs to Model WDR62‐Related Microcephaly

**Figure 3:**
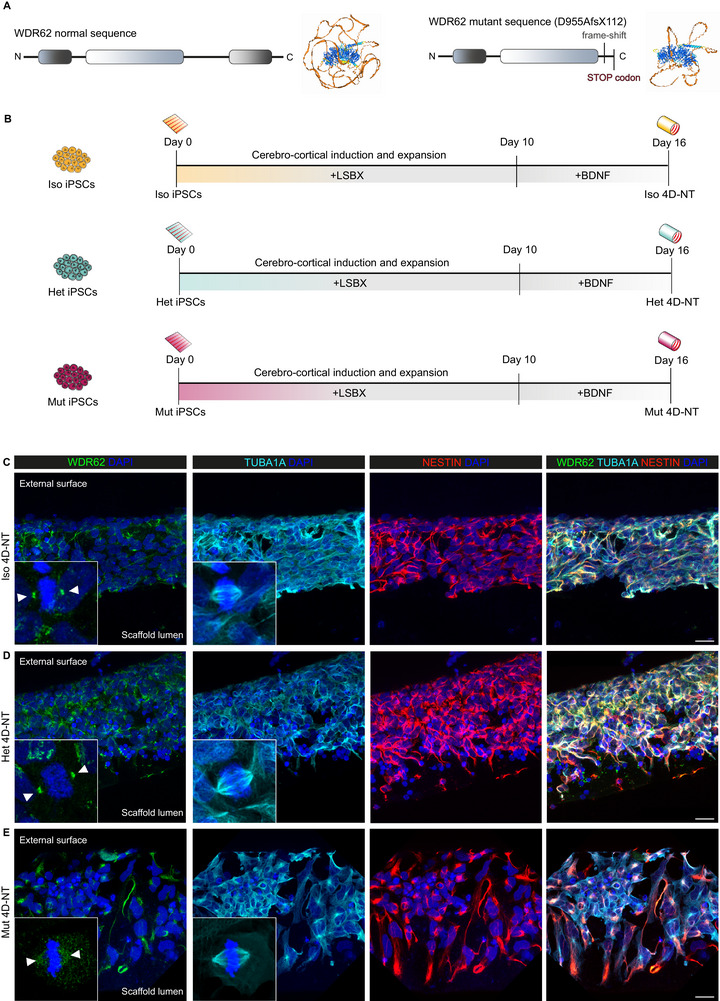
Using 4D‐NTs to model WDR62‐associated microcephaly (MCPH2). A) Depiction of the native and mutant WDR62 protein harboring an aberrant 112 aminoacid tail and a premature STOP codon at the C‐terminus. With respect to the native conformation, the 3D reconstruction^[^ ^33^, ^34^ ^]^ underlines an altered tertiary structure. B) Iso, Het, and Mut iPSCs are used to generate Iso, Het, and Mut 4D‐NTs via a Dual SMAD inhibition‐based protocol (LSBX, for LDN193189, SB431542, XAV939). C–E) Representative confocal images of Iso, Het, and Mut 4D‐NT sections. Immunofluorescence analysis shows WDR62 localized to the TUBA1A^+^ spindle poles in mitotic NPCs from Iso and Het 4D‐NTs, while is dispersed during mitosis in Mut 4D‐NT NPCs. Scale bars: 20 µm in C, D, E, and 5 µm in insets. Nuclei are counterstained with DAPI.

Next, asked whether the 4D‐NT platform can be used to investigate the mechanisms underlying genetic disorders of neurodevelopment, e.g., malformations of cortical development. We took advantage of iPSCs derived from an individual with *WDR62*‐associated MCPH, with a homozygous D955AfsX112 mutation^[^ ^27^, ^28^ ^]^ (Mut, hereafter D955), from an unaffected parent heterozygous for the mutation (Het), and the corresponding isogenic corrected CTRL line (Iso)^[^ ^27^ ^]^ (**Figure** **3**A,B). As shown in Figure 3A, the mutation leads to the loss of the C‐terminal part of the WDR62 protein, producing a shortened protein, as highlighted in the 3D reconstruction through AlphaFold^[^ ^38^, ^39^ ^]^, with a 112 amino acid tail that is not present in the native sequence. Iso, Het, and Mut iPSCs were employed to generate Iso, Het, and Mut 4D‐NTs, respectively (Figure 3B).

The D955A mutation has been previously found to impact WDR62 localization, primary cilia, and cell cycle progression in NPCs.^[^ ^27^ ^]^ In particular, the mutation prevents canonical WDR62 localization to the spindle poles during mitosis^[^ ^40^ ^]^ in patient fibroblasts and in Mut iPSCs,^[^ ^27^, ^28^ ^]^ contrasting with Iso and Het iPSCs. Moreover, in Mut mitotic iPSCs, WDR62 was retained at the Golgi apparatus.^[^ ^27^ ^]^ Thus, we asked whether these altered phenotypes were present in the 4D‐NT model. To do this, Iso, Het, and Mut iPSCs were applied to 4D‐NTs. On Day 16, in mitotic NPCs, WDR62 was localized to the TUBA1A^+^ spindle poles in Iso and Het 4D‐NTs, but not in Mut 4D‐NTs, where the protein was dispersed (Figure 3C–E).

**Figure 4:**
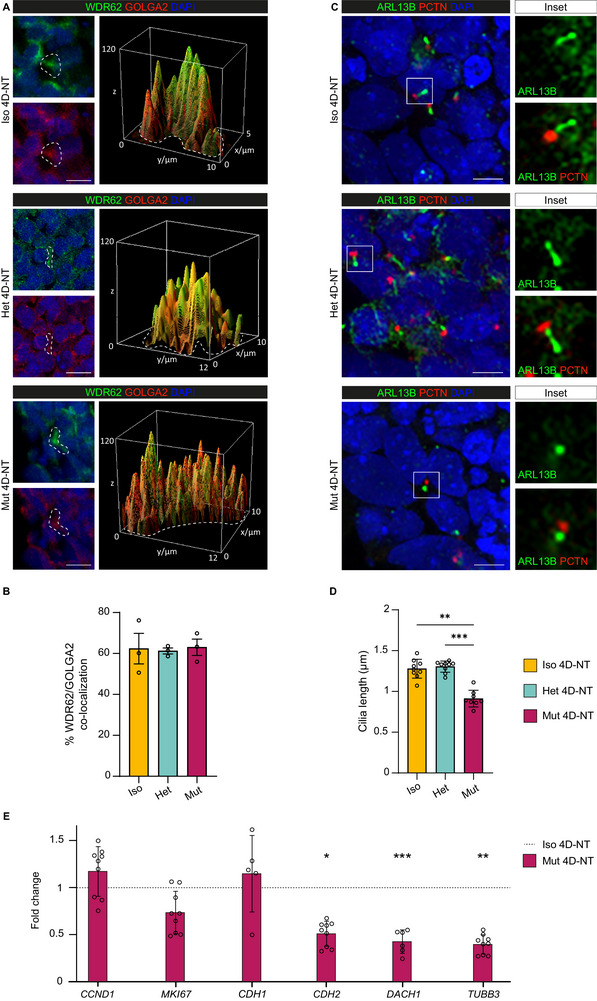
Mut 4D‐NTs recapitulate the main hallmarks of WDR62‐related MCPH. A) Representative confocal images and 3D surface plots of the dotted areas of WDR62 and Golgi apparatus (GOLGA2^+^) co‐localization during interphase in Iso, Het, and Mut 4D‐NTs. B) Co‐localization analysis shows high WDR62/GOLGA2 co‐occurrence in Iso, Het, and Mut 4D‐NT NPCs with no significant differences between genotypes. Data are represented as mean ± SD, with dots showing single‐section data points. Replicates *n* = 3 per genotype, total objects *n* = 90; One‐way ANOVA. C) Representative confocal images of primary cilia stained with the ARL13B marker and corresponding PCTN^+^ basal bodies in Iso, Het, and Mut 4D‐NT NPCs. D) Quantitative analysis of primary cilia length (measured from the tip to the base of the cilium) shows significantly shorter primary cilia in Mut 4D‐NTs compared with Iso and Het 4D‐NTs. Data are represented as mean ± SD, with dots showing single‐section data points. Replicates *n* = 9 per genotype, total cells *n* = 810; Kruskal‐Wallis test. E) Quantitative analysis of mRNA expression levels detected by RT‐qPCR of Mut 4D‐NT samples compared with Iso 4D‐NTs shows no differences in genes associated with NPC proliferation but highlights reduced neuralization. Data are represented as mean ± SD, with dots showing the mean of a technical triplicate and are shown as fold change in mRNA expression relative to *GAPDH*, a housekeeping gene, according to the 2^−ΔΔCT^ method. 4D‐NT samples *n* = 9; unpaired t‐test. ^*^ *p*‐value < 0.05, ^**^ *p*‐value < 0.01, and ^***^ *p*‐value < 0.001; Scale bars: 10 µm in A and 5 µm in C. Nuclei are counterstained with DAPI.

We then investigated the subcellular localization pattern of WDR62 in Iso, Het, and Mut 4D‐NT interphase NPCs (**Figure** **4**A). We observed WDR62 at the Golgi apparatus, similarly to what we reported in NPCs, growing as a monolayer, and in cortical organoids.^[^ ^27^ ^]^ Immunofluorescence and WDR62/GOLGA2 co‐localization analysis showed WDR62 highly localizing to the Golgi apparatus during interphase in Iso, Het, and Mut 4D‐NT NPCs, thus confirming the previously reported localization pattern and further validating the 4D‐NT model (Figure 4B).

WDR62 is also involved in the biogenesis of primary cilia, antenna‐like structures that allow NPCs to sense and convey extracellular cues and to orchestrate self‐renewal and differentiation during neural development.^[^ ^41^, ^42^ ^]^ For this, we immunostained primary cilia with ARL13B and basal bodies with PCTN. Quantitative analysis revealed shorter primary cilia in Mut 4D‐NTs compared with Iso and Het counterparts (Figure 4C,D), confirming the previous observations.^[^ ^27^, ^43^ ^]^

Finally, we evaluated cell cycle by analyzing the expression of *CCND1* (Cyclin D1) and *MKI67* (Marker of proliferation Ki67), and the neuralization level by analyzing the expression of *CDH1* and *CDH2*, *DACH1* (Dachshund family transcription factor 1), a marker of early NPCs in the neural tube,^[^ ^44^ ^]^ and *TUBB3*, expressed in differentiating neurons (Figure 4E).

From this analysis, we found that Mut 4D‐NTs showed reduced neuralization and lower expression of markers typical of NPCs and neurons.

Altogether, these data validate multiple WDR62 phenotypes on the 4D‐NT system, a valuable tool applicable to investigating early cellular abnormalities in malformations of cortical development.

## 3 Discussion

Advances in cellular technologies over the last decades have made it possible to generate pluripotent stem cells from somatic sources through cellular reprogramming. This discovery has been instrumental for advancing in human brain development research by providing in vitro access to time windows and processes otherwise hard to access.^[^ ^45^, ^46^ ^]^ More recently, the combined use of iPSCs and microfluidic devices has led to the development of 3D‐patterned neural tube‐like structures.^[^ ^15^, ^47^ ^]^ However, their static structure does not resemble the dynamics typical of human developing tissues. The advent of 4D bioprinting allows for precisely manufactured biocompatible active scaffolds that can change their shape over time upon exposure to predefined external stimuli and are designed to interact with cells, thus more closely mimicking tissue dynamics. Here, combining iPSCs and 4D bioprinting, we describe the generation and characterization of human 4D‐NTs to model normal and defective neural tube development.

Congenital neurodevelopmental disorders are severe conditions. In humans, clinical reports suggest a multifactorial polygenic or monogenic origin, highlighting the importance of gene‐gene and gene‐environment interactions.^[^ ^3^, ^48^, ^49^ ^]^ Among these disorders, primary microcephaly is a particularly devastating condition that leads to abnormal brain development, affecting both brain architecture and cellular composition, as well as cognitive function.^[^ ^21^ ^]^

Despite animal models capturing some aspects of these neurodevelopmental defects, including disruption of cytoskeleton, cell cycle, and molecular regulation of cell viability, some human‐specific genetic traits are not fully recapitulated.^[^ ^8^, ^9^, ^10^, ^11^, ^12^, ^13^ ^]^ In vitro models, such as iPSCs and their derivatives, represent a crucial approach in the biomedical field. In recent years, innovative platforms have been developed to more accurately replicate underlying pathophysiological mechanisms. However, they still have some limitations, such as the inability of 2D cultures to fully recapitulate the complex architectural aspects of the developing CNS. More recently, 3D organoid and assembloid systems, which incorporate a variety of cell types and thus more closely mimic tissue organization, have been established. However, the main limitations are represented by their intrinsic variability and by the differential exposure of their inner versus outer regions to nutrients and oxygen that may impact their viability and homogeneity.^[^ ^7^, ^50^ ^]^ Nonetheless, for the study of a specific stage of neural development, such as neural tube formation, additional methodologies have been developed.^[^ ^15^, ^51^ ^]^ The majority involve external supports to induce the tubular structure and patterning (namely, Matrigel embedding, bioengineered chips, and microfluidic devices).^[^ ^15^, ^52^, ^53^, ^54^, ^55^ ^]^ Despite the numerous benefits, some critical issues remain, as both organ‐on‐chip systems and 2D‐micropatterned approaches offer controlled and functional forms, yet lack the self‐organization seen in natural development. In addition, the cost of bioengineered devices is not negligible.

Here, we characterized and validated a novel platform that potentially overcomes the constraints related to cost, efficacy, differential viability, and reproducibility: a dynamic 4D scaffold (namely 4D‐NT), which combines stem cell technology with 4D printing with smart materials. This tool is adaptable for various purposes in the neurodevelopment and biomedical fields, since it enables printed structures to mimic structural and functional processes, containing a layer of NPCs radially arranged around a cylindrical lumen. 4D‐NTs are responsive to water‐based stimuli and incorporate time as a new, fourth dimension. Thus, the gelatin‐GPTMS‐based systems we characterized (i.e., 2D‐uNTs and 4D‐NTs) demonstrated permissive, and even enhanced, neuroinductive capabilities compared to the canonical 2D neural differentiation model (i.e., 2D‐NPCs). The Side I of 4D scaffolds consists of GPTMS‐GEL‐15 and printed stripes of GPTMS‐GEL‐5, and no variations in cell morphology or behavior were observed on the different parts.

Furthermore, 4D‐NTs exhibit the ability to self‐fold and acquire a stable neural tube‐like shape. These features represent the major advantages of the 4D‐NT system over conventional 2D and 3D systems, enabling the creation of ductile, responsive, and engineerable in vitro models that more closely resemble the developing neural tube.

Additionally, when compared to the developing human neural tube, the 4D‐NT showed, even though not homogeneously, high biologically relevant properties, including size and NPC radial orientation. To demonstrate that the 4D‐NT platform coupled with iPSC technology constitutes a powerful tool to investigate neurodevelopmental disorders, we applied the system to a previously characterized iPSC‐based model of *WDR62‐*related microcephaly. Mut 4D‐NTs closely recapitulated the pathological hallmarks found in NPCs, including impaired WDR62 spindle poles to Golgi apparatus shuttling and shortening of primary cilia, described in 2D‐NPCs and cerebral organoids.^[^ ^27^ ^]^ Mut 4D‐NTs also showed general reduced neural induction that may deserve further analysis, as it aligns with the patient's phenotype.^[^ ^56^ ^]^

Collectively, this study provides a proof‐of‐concept for the establishment of the 4D‐NT model that represents a novel tool for dissecting normal human neurodevelopment and neurological disorders. We anticipate that the system can be further developed, particularly in extending the duration of self‐folding, potentially achievable through the use of slower water‐absorbing materials, and in enhancing the uniformity of NPC polarity across the entire 4D‐NT surface via functionalization.

Nonetheless, this 4D‐NT system lays the basis for reliable and reproducible alternatives to traditional 2D and 3D in vitro models for studies on human brain development and disease.

## 4 Experimental Section

### Ethics Statement

All cell work was performed according to National Institutes of Health (NIH) guidelines for the acquisition and distribution of human tissue for biomedical research purposes and with approval by the Human Investigation Committee and Institutional Ethics Committee of each institution from which the samples were obtained (University of Pisa Review No. 29/2020 and Yale No. 9406007680). Appropriate informed consent was obtained, and all available non‐identifying information was recorded for each specimen. The tissue was handled in accordance with the ethical guidelines and regulations for the research use of human brain tissue set forth by the NIH and the WMA Declaration of Helsinki. The human brain tissue sections used in this study derive from postmortem specimens obtained by the Human Developmental Biology Resource (Project approval 200578).

### iPSC Culture and Differentiation

HSB 311 iPSCs #1 (CTRL_1_) and HSB 314 iPSCs #47 (CTRL_2_) were obtained from skin fibroblasts as previously described.^[^ ^29^, ^30^ ^]^ Patient and parent iPSCs were respectively derived from a microcephalic individual carrying a homozygous (D955A) mutation in *WDR62* (Mut) and from its unaffected parent (Het) carrying the *WDR62* mutation in heterozygosity by reprogramming patient and parent‐derived fibroblasts.^[^ ^28^ ^]^ Isogenic corrected (Iso) iPSCs were then derived through CRISPR‐Cas9 genome editing of Mut iPSCs, obtaining bi‐allelic sequence restoration as described in Dell'Amico et al.^[^ ^27^ ^]^

Human iPSCs were cultured as previously described.^[^ ^27^ ^]^ Briefly, cells were seeded on Matrigel‐ (1:60, Corning; #356234) or rhLaminin‐521‐ (10 µg mL^−1^, Thermo Fisher Scientific; #A29248) coated culture plates and maintained in Stem Flex Basal medium (Thermo Fisher Scientific; #A3349201). At 70% confluence, cells were passaged with EDTA (0.5 mm) at room temperature (RT). After 3–5 min of incubation, the EDTA solution was removed, and cells were gently detached from the dish with a small volume of medium, generating clumps of six to eight cells. In standard conditions (37°C, 5% CO_2_), iPSC colonies typically grow within 4–5 days.

For cerebro‐cortical differentiation, iPSCs were exposed to an optimized Dual SMAD inhibition protocol.^[^ ^27^ ^]^ Briefly, iPSCs were detached at a single‐cell level by means of Accutase (Corning; #25058) pre‐warmed at 37°C. After 3 min of incubation, the enzyme was inactivated with 4 volumes of PBS. Cells were then centrifuged at 200 x g for 3 min. After pellet resuspension, cells were counted and plated at a density of 0.7 × 10^5^ cells cm^−2^ in neural induction medium (DMEM‐F12/Neurobasal mixture 1:1, 1% N2, 2% B27, 20 µg mL^−1^ insulin, 1% MEM‐nonessential amino acids, 1% L‐glutamine, 0.1% 2‐mercaptoethanol) supplemented with Dual SMAD inhibition factors, i.e., 10 µm SB431542 (TargetMol; #T1726), 100 nm LDN193189 (STEMCELL Technologies; #72144), 2 µm XAV939 (STEMCELL Technologies; #72674), and 10 µm Y‐27632 (TargetMol; #T1725) to increase cell survival. Medium was replaced daily for 10 days. On Day 10, medium was partially substituted (50% of the total volume) with neural differentiation medium (DMEM‐F12/Neurobasal mixture 1:1, 1% N2, 2% B27, 1% L‐glutamine) supplemented with 30 ng mL^−1^ BDNF (R&D Systems; #248‐DB‐025). From Day 10, medium was partially replaced every 3 days (half medium plus 20% volume to compensate for the evaporation rate), and cell samples were fixed or collected for RNA extraction on Day 16.

### Smart Scaffold Fabrication for 4D‐NT Generation

4D smart scaffolds were fabricated as described in Chiesa et al.^[^ ^31^ ^]^ Briefly, Type A gelatin from porcine skin (Sigma–Aldrich; #G2500), cross‐linked by GPTMS ((3‐glycidoxypropyl)trimethoxysilane; Sigma–Aldrich; #4401) was selected as bulk material. In detail, the 4D smart scaffolds were made of two layers of GPTMS cross‐linked gelatin at different concentrations (the concentration of the two layers was tuned by swelling characterization, see Supporting Information). The high‐swelling layer of the scaffold was made with a 15% w/v gelatin in phosphate‐buffered saline solution 1X (PBS, Sigma–Aldrich; #P3813) cross‐linked with 92 µl g^−1^ of GPTMS (GPTMS‐GEL‐15), whereas the low‐swelling layer was made with a 5% w/v gelatin solution in PBS 1X cross‐linked with 368 µl g^−1^ of GPTMS (GPTMS‐GEL‐5). Then, a film of GPTMS‐GEL‐15 was fabricated by casting it into a Petri dish. After that, three‐layered stripes of GPTMS‐GEL‐5 were bioprinted on the GPTMS‐GEL‐15 film via piston‐driven extrusion‐based bioprinting (printing parameters are reported in the Supporting Information). Specifically, the surface area of the GPTMS‐GEL‐5 stripes accounted for 30% of the total 4D scaffold surface area of Side I, while the remaining 70% was composed of GPTMS‐GEL‐15 (further details are reported in “Supporting Information”). Lastly, samples were dried for 48 h at RT and finally cored into 10 mm x 5 mm rectangular samples, with the bioprinted stripes parallel to the short side.

### Scaffold Fabrication for 2D‐uNT Generation

Static monolayer 2D scaffolds entirely made of GPTMS‐GEL‐15 solution were fabricated by solvent casting, followed by air drying at room temperature for 24 h.

### iPSC Seeding on Scaffolds—4D‐NT Generation

Prior to seeding, 4D scaffolds were coated with Matrigel 1:50 diluted in ice‐cold PBS. To avoid scaffold actuation, a custom holder (made by casting PDMS in an ad hoc fabricated mold as detailed in Supporting Information) was placed on top of the scaffold to allow coating and seeding. Matrigel solution was gently dropped on the scaffold through the holder seeding window, and incubated for 30 min at 37°C. After incubation, excess PBS was removed and, according to the above‐described procedure, iPSCs were plated through the seeding window at 0.7 × 10^5^ cells cm^−2^ density in 50 µl of Stem Flex medium supplemented with 10 µm Y‐27632, to increase cell viability. Cells were then placed in the incubator to allow adhesion. After 1 h, Stem Flex supplemented with 10 µm Y‐27632 was added, and the holder was carefully removed, then allowing scaffold actuation. After 24 h, Stem Flex medium was replaced with neural induction medium (Day 0). 4D‐NTs were then cultured until Day 16 according to the above‐described differentiation protocol.

### iPSC Seeding on Scaffolds—2D‐uNT Generation

To generate the 2D‐uNTs, 2D scaffolds were first coated with a Matrigel solution diluted in ice‐cold PBS 1:50 and gently dropped on the scaffold through the holder seeding window. After incubation (30 min at 37°C), excess PBS was removed, and iPSCs were seeded at the same density and according to the same procedure followed for the 4D‐NT generation. Cells were then placed in the incubator to allow adhesion. After 1 h, Stem Flex supplemented with 10 µm Y‐27632 was added, and the holder was carefully removed. After 24 h, Stem Flex medium was replaced with neural induction medium (Day 0). 2D‐uNTs were then cultured until Day 16 according to the above‐described differentiation protocol.

Similarly, to generate experimental controls (2D‐NPCs), iPSCs were seeded on standard Matrigel‐coated multiwells (24w‐multiwell plate) at the same density used for 4D‐NTs and 2D‐uNTs.

### Immunofluorescence Assay

4D‐NT, 2D‐uNT, and 2D‐NPC samples: after fixation with 4% formaldehyde (FA) for 20 min at 25°C for 4D‐NT and 2D‐uNT samples or 4% FA for 12 min for 2D‐NPC samples, three washes with PBSX (1% vol/vol TritonX‐100 – Sigma–Aldrich; #T9284‐500ml – in PBS) were performed. Then, samples were incubated at RT in permeabilization solution (0.5% vol/vol TritonX100 in PBS^Ca2+/Mg2+^) for 10 min and then in blocking solution (5% FBS (Thermo Fisher Scientific; #10500064), 0.3% vol/vol TritonX‐100 in PBS^Ca2+/Mg2+^) for 1 h at RT. Then, samples were incubated with primary antibodies in antibody solution (3% FBS, 0.2% vol/vol TritonX‐100 in PBS^Ca2+/Mg2+^) overnight (ON) at 4°C. The next day, samples were washed three times with PBSX before incubation with the corresponding secondary antibodies (all diluted 1:500) and DAPI (Sigma–Aldrich; #32670‐25mg) for 1 h at RT. 2D‐NPC and 2D‐uNT samples were mounted with Aqua‐Poly/Mount (VWR; #87001902) on microscope slides, while 4D‐NT samples were either kept in plates with PBS^Ca2+/Mg2+^ for whole mount confocal imaging or cryopreserved with 30% sucrose ON and embedded in OCT (VWR; #361603e) for cryostat sectioning.

For immunofluorescence analysis, cryostat sections were washed once with PBS for 10 min and permeabilized with 0.5% Triton X‐100 in PBS for 10 min. Sections were then washed in PBS and treated with sodium citrate‐based R‐buffer A (EMS; #62706‐10) in 2100‐Retriever (EMS; #62706) at 120°C for 20 min. After antigen retrieval, sections were washed with PBS and blocked with 5% horse serum (Thermo Fisher; #26050070), 1% bovine serum albumin (BSA) in PBS with 0.3% Triton X‐100 for 1 h at RT. Primary antibodies were diluted in blocking solution and incubated at 4°C for ON (**Table** 1). The following day, sections were washed three times in PBSX. Secondary antibodies and DAPI were diluted in the antibody solution for 1 h at RT. Then, sections were washed twice in PBSX and once in PBS and finally mounted with Aqua‐Poly/Mount on microscope slides for confocal microscopy.

**Table 1:** List of the antibodies and probes used in this study.

Target	Dilution	Cat n.	Brand
ARL13B	1:500	PA5‐61840	Invitrogen
CTNNB1	1:150	BD610153	BD Bioscience
FOXG1	1:500	ab18259	Abcam
GOLGA2	1:200	A‐21270	Invitrogen
Nanog	1:100	09‐0020	Stemgent
Nestin	1:200	MAB1259	RD
OCT4	1:100	09‐0023	Stemgent
PAX6	1:100	901301	Biolegend
PCTN	1:1000	ab28144	Abcam
Phalloidin‐488	1:400	a12379	Thermo Fisher
SOX2	1:400	ab5603	Millipore
TJP1	1:100	617300	Invitrogen
TUBA1A	1:1000	mca77g	Bio‐rad
TUBB3	1:1000	ab41489	Abcam
WDR62	1:500	A301‐560A	Bethyl Lab

Images were acquired using a laser scanning confocal microscope (Nikon AX Ti2) and spinning‐disk confocal microscope (Nikon Crest Optics Cicero Eclipse Ti‐2).

### RNA Extraction, Purification, Retro‐Transcription, and qPCR

After removing the medium, cell samples were washed once with PBS. Then, PBS was removed, and an appropriate amount of RNA Protect Cell reagent (Qiagen; #1038674) was added to cover the samples. After 2 min, 2D‐NPC samples were gently scraped and collected in a 1.5 ml tube, while 4D‐NT and 2D‐uNT samples were directly placed in the 1.5 ml tube with RNA protect Cell reagent. Then, samples were stored at −80°C. For processing, samples were thawed, and 4D‐NT and 2D‐uNT samples were manually homogenized using a micropestle. RNA was extracted and purified with RNeasy spin column (Qiagen; #74104) and RNase‐Free DNase set (Qiagen; #79254) according to the manufacturer's instructions. After quantification, 500 ng of RNA were retro‐transcribed to cDNA using GoScript Reverse Transcription System (Promega; #A5001) according to the manufacturer's instructions. qPCR was performed using QuantStudio 3 Real‐Time PCR System (Applied Biosystems; #A28137) with SensiMix SYBR No‐ROX kit (Meridian BIOSCIENCE; #QT650‐05). Thermal cycling conditions: denaturation at 95°C for 10 min and 40 cycles of 95°C for 15 s and 60°C for 1 min. Data were expressed as fold change of the expression of each gene relative to the *GAPDH* housekeeping gene, according to the 2^−ΔΔCT^ method. Primer list is reported in **Table** 2.

Analyses were performed in biological replicates (n≥3). For each sample, three technical replicates were analyzed.

**Table 2:** List of the primers used for qPCR analysis.

Gene	Forward Primer (5’‐3’)	Reverse Primer (5’‐3’)
*CCND1*	GCGGAGGAGAACAAACAGATC	GAGGGCGGATTGGAAATGAAC
*CDH1*	AATCCCACCACGTACAAGGG	GGTATTGGGGGCATCAGCAT
*CDH2*	TGCATGAAGGACAGCCTCTTC	GCTTCTCACGGCATACACCA
*COL4A1*	AAGGGCGACAGAGGTTTGC	ATAAAACTCACCAGGCTCCCC
*CTNNB1*	CTTCACCTGACAGATCCAAGTC	CCTTCCATCCCTTCCTGTTTAG
*DACH1*	GTGGAAAACACCCCTCAGAA	CTTGTTCCACATTGCACACC
*FOXG1*	TGGACGCAGACCTTGAGAAC	GGGCACCTTTACTACGAATGC
*MKI67*	CTTTGGGTGCGACTTGACG	TACAACTCTTCCACTGGGACG
*NESTIN*	CAGGGGCAGACATCATTGGT	CATTCCTTGCCCCACTTCCT
*NID1*	ACTGCGTGGACAAGATGGTT	ATGATGGTGGTTGGCTCTCC
*OTX2*	GGCACTGAAAATCAACTTGCCC	CTGTTGTTGGCGGCACTTAG
*TJP1*	TTTGGTGATGTGTGGTCCCC	AGACACTTGTTTTGCCAGGTTT
*TUBB3*	GGCAACTACGTGGGCGAC	GCACGTACTTGTGAGAAGAGGC
*VIM*	GCTAACCAACGACAAAGCCC	GATTGCAGGGTGTTTTCGGC

### Orientation Analysis

Confocal images showing the distribution of nestin^+^ cells (see Figure S7, Supporting Information) were analyzed to quantify cell orientation with the *Directionality* plug‐in (ImageJ). Briefly, the plug‐in allows for computing a histogram with 90 bins indicating the number of structures (cells) in a given angular direction, from −90° to +90°.^[^ ^32^ ^]^ The images were re‐oriented prior to processing in order to assign a common 0° point. Then, the data were averaged and plotted.

### Co‐Localization Analysis

Following immunofluorescent staining on 4D‐NT sections, co‐localization analysis for WDR62 and GOLGA2 signal was performed using the JACoP plugin^[^ ^33^ ^]^ (ImageJ) on 500 nm Z‐stack confocal images. This plugin allows for calculating the percentage of co‐localization through Manders’ coefficient between two selected objects, measuring the co‐occurrence (on three dimensions) of every single pixel unit – defined by size through threshold setting – constituting the object itself. To calculate WDR62‐GOLGA2 co‐localization in 4D‐NT sections, the WDR62 signal was selected within a region of interest (ROI) in channel 1 (WDR62) and channel 2 (GOLGA2) composites. Then, the co‐occurrence of channel 1 and channel 2 pixels was calculated in single Golgi objects.^[^ ^27^ ^]^

### Cilia Length Analysis

Following immunofluorescent staining on 4D‐NT sections, Z‐stacks with a step size of 500 nm were acquired to create Z‐projections (maximum intensity projection) of primary cilia for each genotype. Single cilium length was measured with the ‘NeuronJ’ plugin (ImageJ)^[^ ^34^ ^]^ as described in Dell'Amico et al.^[^ ^27^ ^]^ Starting at the midpoint of the first pixel on the perinuclear side of the cilium, a line was manually traced along the ciliary axis until the midpoint of the last pixel at the opposite end was reached.

### Statistical Analysis

Data were presented as mean ± SD unless described otherwise. All experiments were performed in biological replicates n ≥ 3 (rounds of differentiations/batches). The size of the population (N) was reported for each experiment in the corresponding figure legend. Counts and analyses were performed blinded to conditions and genotypes. Images were acquired with a Nikon AX Ti2 Confocal Microscope, Nikon Crest Optics Cicero Eclipse Ti‐2 spinning disk confocal microscope, and NIS‐Elements C and NIS‐Element AR (Ver. 5.40 and Ver. 6.10.01) 64‐bit software. Confocal images were then processed and quantified with ImageJ.

## Conflict of Interest

The authors declare no conflict of interest.

## Author Contributions

C.D.A. and I.C. equally contributed to the work. C.D.A. contributed to data curation, formal analysis, investigation, and writing of the original draft. I.C. and A.E. were responsible for the manufacture of 2D and 4D samples and data analysis. A.T. performed formal analysis and investigation. P.M. and C.M. conducted spinning disk confocal imaging and analysis. A.L. handled writing, review and editing. C.D.M. oversaw conceptualization, funding acquisition, and manuscript revision, and M.O. provided conceptualization, supervision, funding acquisition, writing, review, and editing.

## Supporting information



Supporting Information

Supplemental Video 1

## Data Availability

The data that support the findings of this study are available from the corresponding author upon reasonable request.
