# Supplementary material for: A Human Neural Tube Model Using 4D Self‐Folding Smart Scaffolds
Source: Adv Healthc Mater. 2025 Oct 23;15(5):e01405. doi: 10.1002/adhm.202501405 (PMC12864576; doi:10.1002/adhm.202501405)
Supplement: Supplementary file 1 — Supporting Information [file ADHM-15-0-s002.pdf]

ADVANCED HEALTHCARE MATERIALS

Supporting Information

for *Adv. Healthcare Mater.*, DOI 10.1002/adhm.202501405

A Human Neural Tube Model Using 4D Self-Folding Smart Scaffolds

Claudia Dell'Amico, Irene Chiesa, Angela Toffano, Alessio Esposito, Piera Mancini, Chiara Magliaro, Angeliki Louvi, Carmelo De Maria and Marco Onorati**

Supporting Information

A human neural tube model using 4D self-folding smart scaffolds

Claudia Dell'Amico, Irene Chiesa, Angela Toffano, Alessio Esposito, Piera Mancini, Chiara Magliaro, Angeliki Louvi, Carmelo De Maria*, Marco Onorati*

C. Dell'Amico: University of Pisa, Department of Biology, Unit of Cell, Molecular and Developmental Biology, and Department of Clinical and Translational Medicine, Pisa, Italy.
I. Chiesa: University of Pisa, Department of Information Engineering and Research Center Enrico Piaggio, Pisa, Italy.
A. Toffano: University of Pisa, Department of Biology, Unit of Cell, Molecular and Developmental Biology, Pisa, Italy.
A. Esposito: University of Pisa, Department of Information Engineering and Research Center Enrico Piaggio, Pisa, Italy.
P. Mancini: University of Pisa, Department of Information Engineering and Research Center Enrico Piaggio, Pisa, Italy.
C. Magliaro: University of Pisa, Department of Information Engineering and Research Center Enrico Piaggio, Pisa, Italy.
A. Louvi: Yale School of Medicine, Department of Neurosurgery and of Neuroscience, New Haven, United States.
C. De Maria: University of Pisa, Department of Information Engineering and Research Center Enrico Piaggio, Pisa, Italy, carmelo.demaria@unipi.it.*
M. Onorati: University of Pisa, Department of Biology, Unit of Cell, Molecular and Developmental Biology, Pisa, Italy, marco.onorati@unipi.it.*

C. Dell'Amico and I. Chiesa equally contributed to the work.

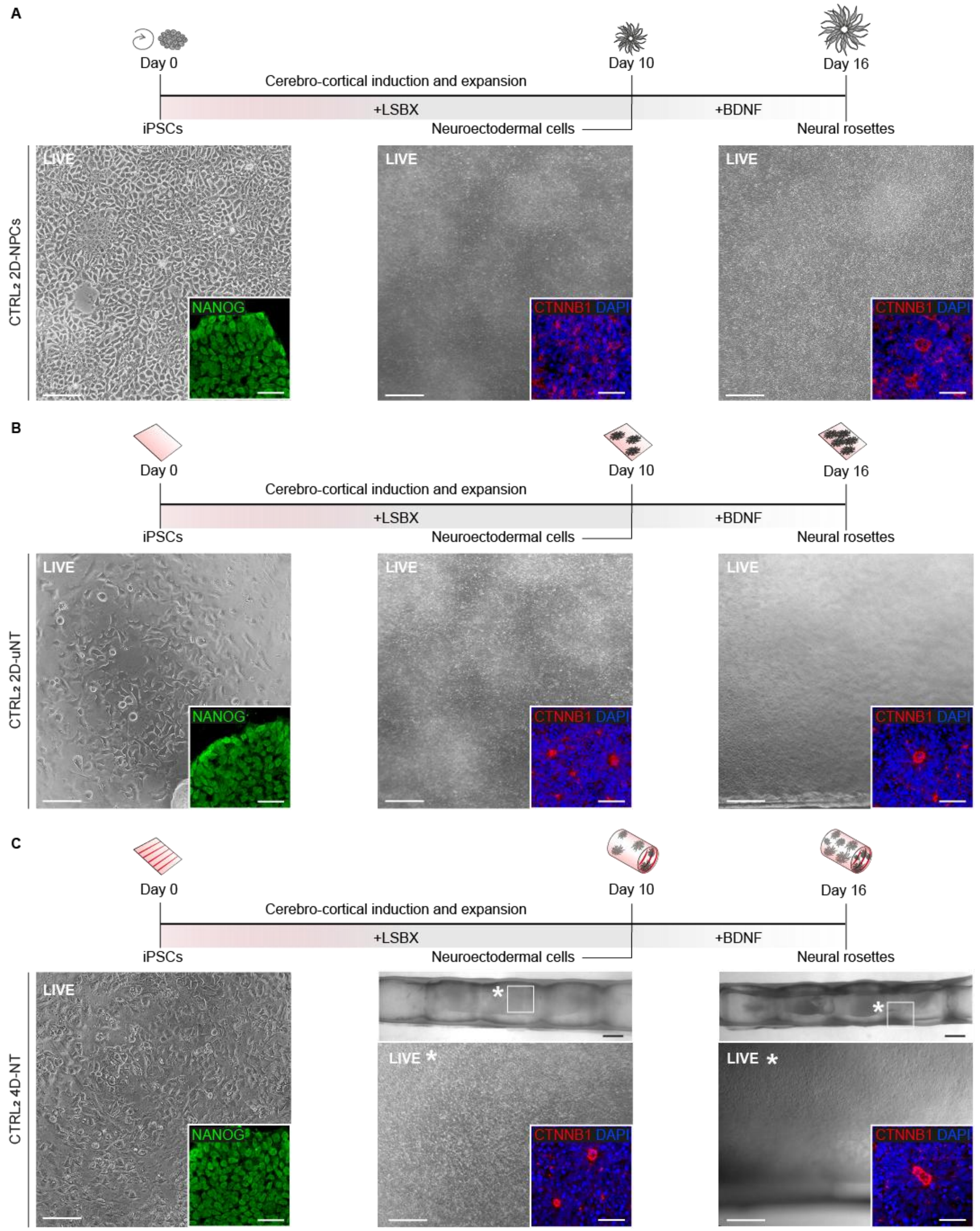

Figure S1: Characterization of 2D and 4D neuroderivatives with the CTRL$_2$ iPSC line. A-C) Representative bright field (LIVE) and confocal immunofluorescence images for Nanog, marking pluripotent stem cells, and CTNNB1, highlighting neural rosettes from CTRL$_2$ iPSC differentiation into 2D-NPCs (A), unfolded neural tube, 2D-uNTs (B), and 4D-NTs (C) conditions. After seeding, iPSCs are driven to a neural fate via a Dual SMAD inhibition-based protocol (LSBX, for LDN193189, SB431542, XAV939). Within 10 days, cells acquire

neuroectodermal identity showing neural rosette formation. From Day 10 onward, cultures are exposed to BDNF and differentiated into neural progenitor cells (NPCs), organized in polarized rosettes. Scale bars: 50 μm in live images (1.5 mm in live images in C, mid and right top zoom out), and 10 μm in insets. Nuclei are counterstained with DAPI.

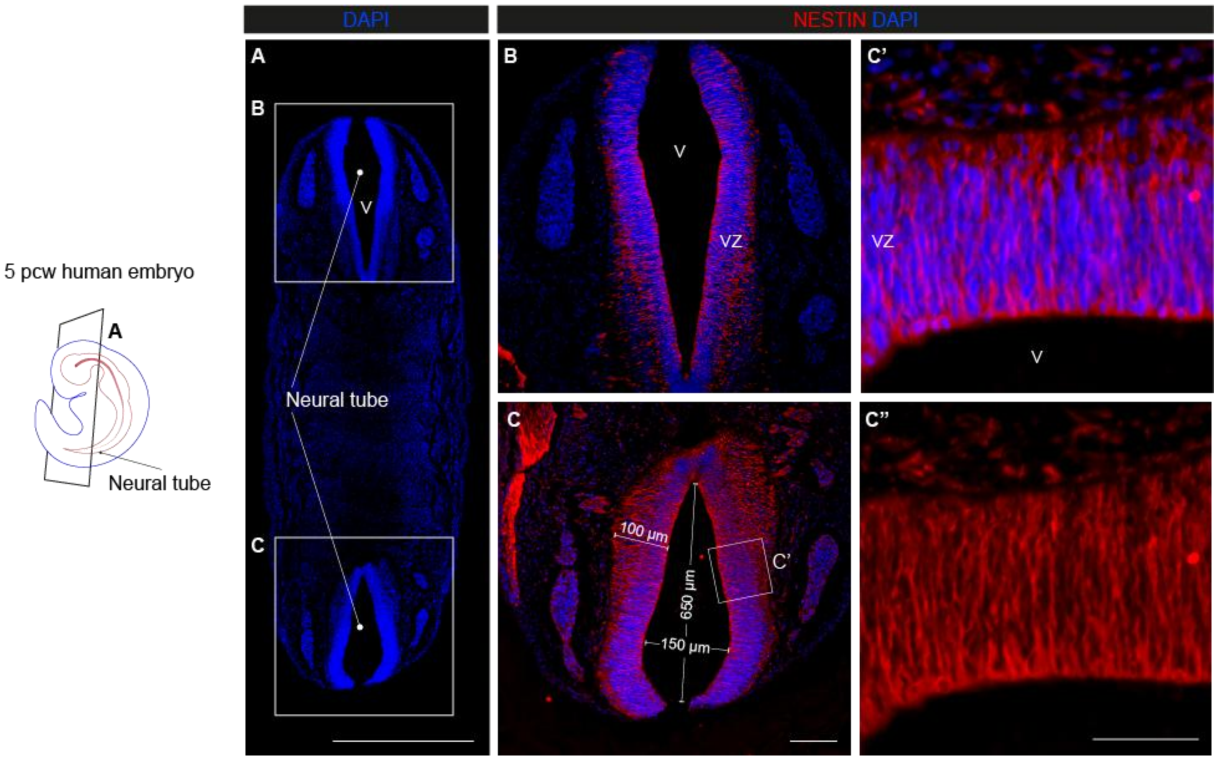

Figure S2: Morphological and tissue organization of the developing human neural tube. Depiction of the 5 pcw human embryo, showing the plane of the section in A. A) Coronal section of a 5 pcw human embryo. The section shows a closing neural tube with a central ventricle (V). B) The developing neural tube is constituted mainly of NPCs (nestin^{+}) organized in an around 100 µm-tick pseudostratified neuroepithelium (i.e., ventricular zone, VZ), radially oriented, perpendicular to the ventricular rim (magnified and 90° rotated area in C' and C''). Scale bars: 500 µm in A, 100 µm in B and C; 50 µm in C' and C''. Nuclei are counterstained with DAPI.

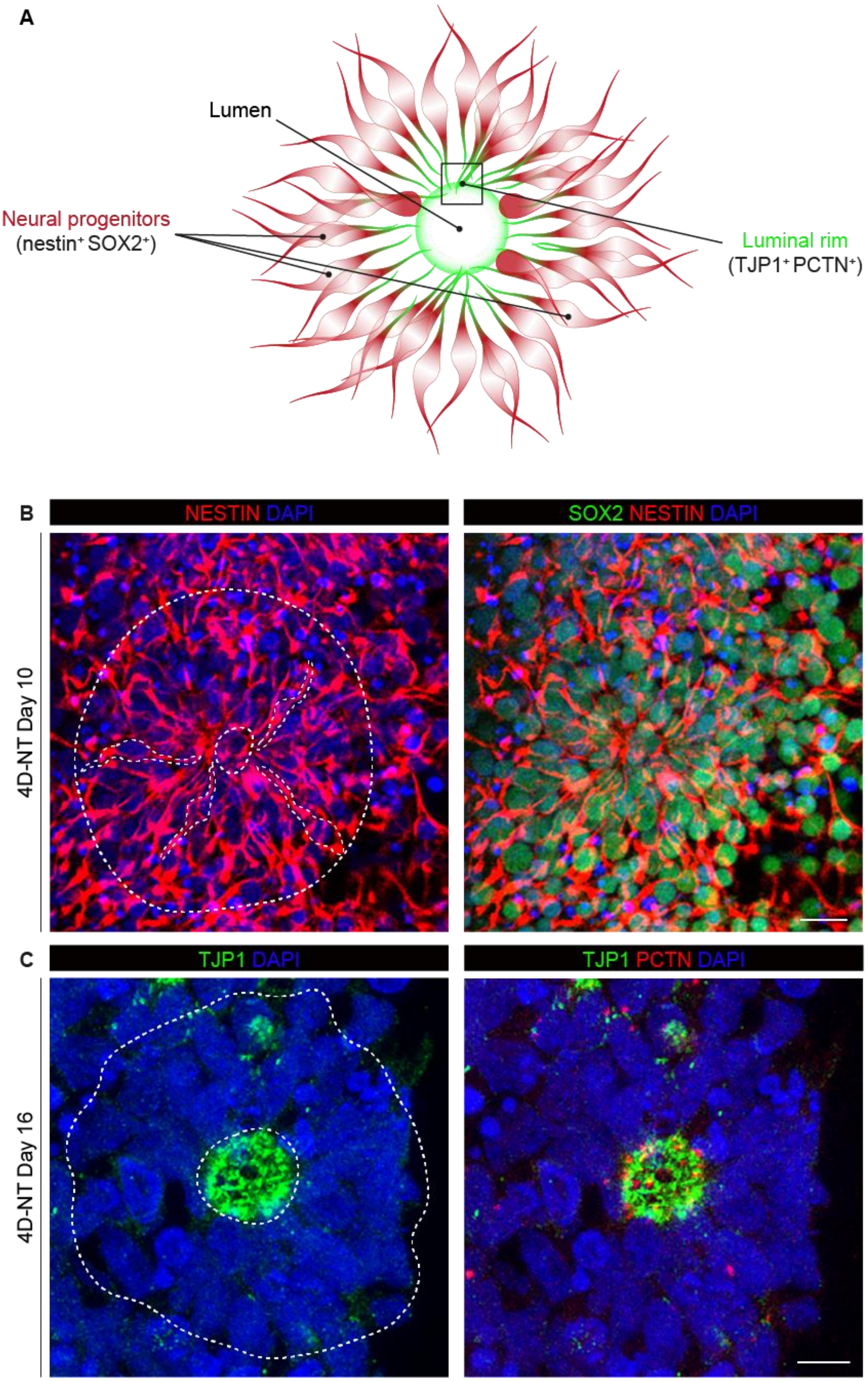

Figure S3: 4D-NTs extensively support neural rosette formation. A) Schematic of NPCs organized in neural rosettes. NPCs (nestin^{+} SOX2^{+}) are apico-basally oriented toward the rosette lumen. The luminal area is TJP1^{+} (zonula occludens) and PCTN^{+} (basal bodies). B) Representative confocal image of nestin^{+} and SOX2^{+} NPCs organized in a neural rosette on Day 10 in 4D-NTs. C) Representative confocal image of neural rosette on Day 16 on 4D-NTs. Rosette structure and luminal rim are highlighted with dotted lines. Scale bars: 20 µm in B, and 10 µm in C. Nuclei are counterstained with DAPI.

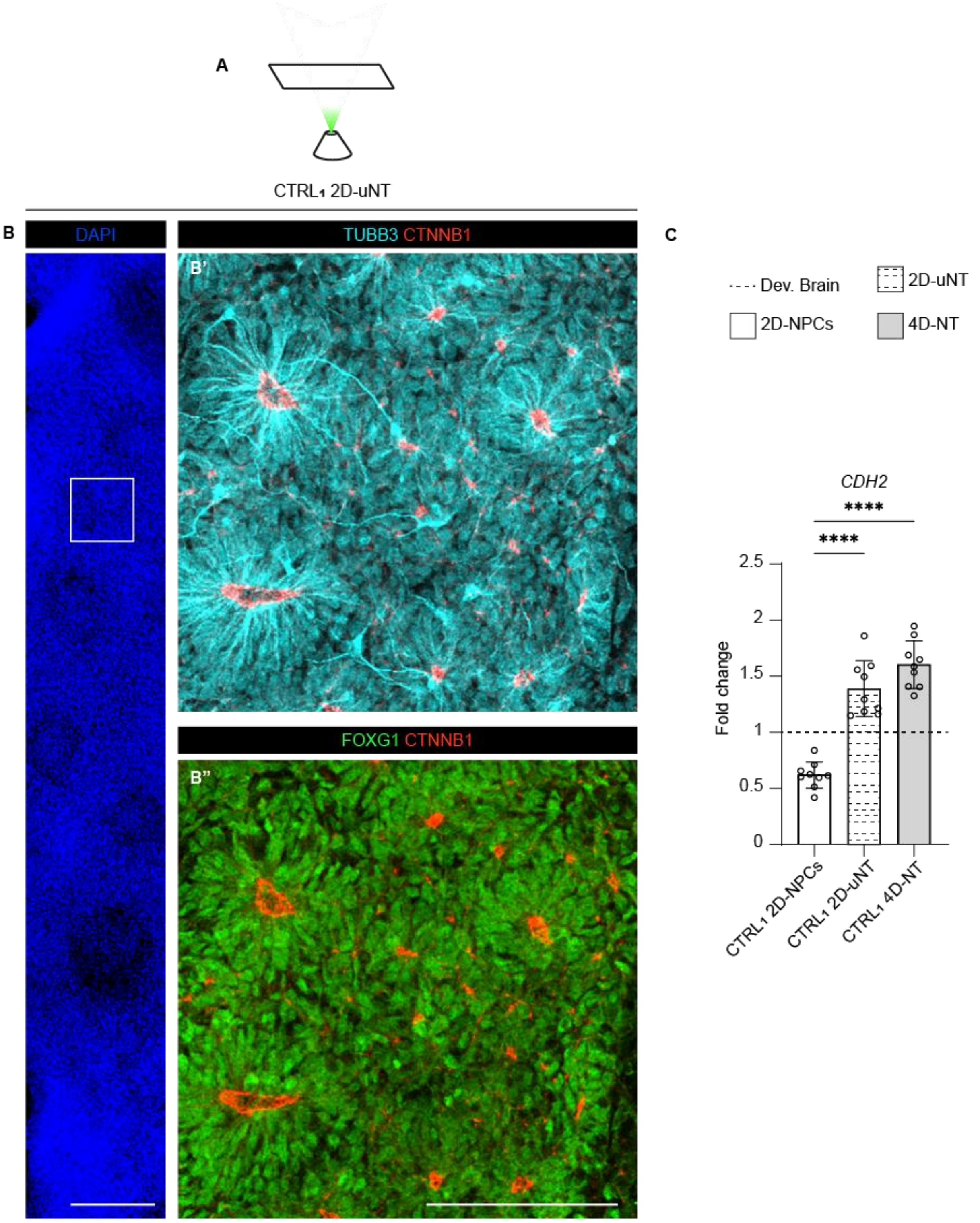

Figure S4: Characterization of Day 16 scaffold models from CTRL$_1$ iPSCs. A) Schematic of the unfolded neural tube (2D-uNT) orientation for confocal imaging. B) On Day 16, 2D-uNT scaffolds are homogeneously populated by NPCs. Magnified area (white square) with TUBB3 and CTNNB1 staining shows fields of neural rosettes distributed on the 2D-uNT, indicating

extended neuralization (B'). NPCs are positive for FOXG1, a telencephalic neuroprogenitor marker (B''). C) Quantitative analysis of mRNA levels detected by RT-qPCR of *CDH2* (N-cadherin) highlights the neuralization level in the 2D-uNT and 4D-NT samples, with respect to the 2D-NPC control. "Dev. Brain" represents a reference of a cDNA pool of developing human brain tissue. Data are represented as mean ± SD, with dots showing the mean of a technical triplicate and are shown as fold change in mRNA expression relative to *GAPDH*, a housekeeping gene, according to the $2^{-\Delta\Delta CT}$ method. $CTRL_1$ samples n=9; ****p-value < 0.0001; One-way ANOVA. Scale bars: 500 µm in B, and 100 µm in B' and B". Nuclei are counterstained with DAPI.

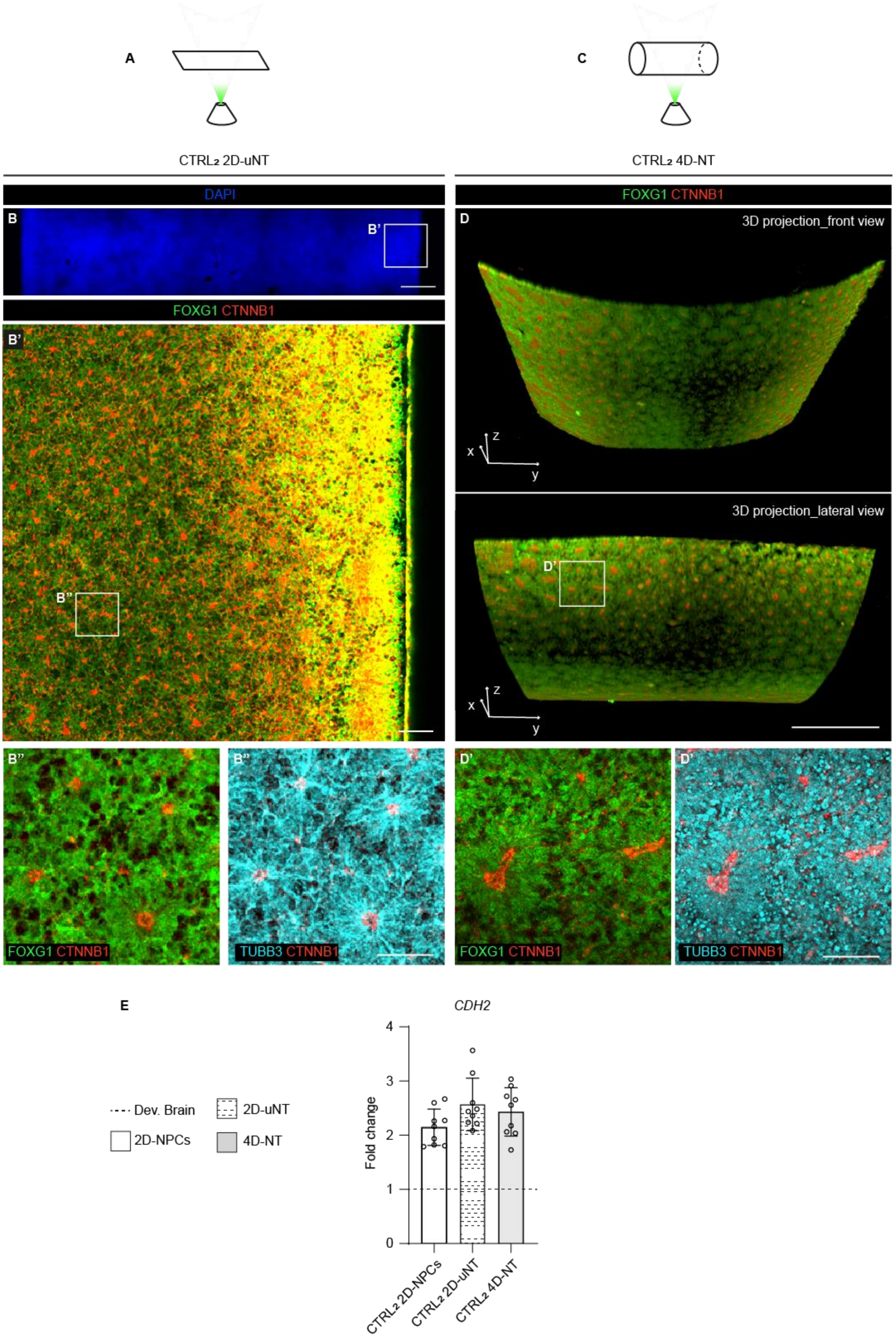

Figure S5: Characterization of Day 16 scaffold models from CTRL$_2$ iPSCs. A) Schematic of the unfolded neural tube (2D-uNT) orientation for confocal imaging. B) On Day 16, 2D-uNT scaffolds are homogeneously populated by TUBB3$^+$ NPCs with telencephalic identity

(FOXG1$^+$) organized in neural rosettes (CTNNB1 staining), as highlighted in B' and B''. C) Schematic of the 4D-NT orientation for confocal imaging. D) 3D projection of the proximal portion to the objective of the 4D-NT (front and lateral view). FOXG1$^+$/CTNNB1$^+$ NPCs are organized into rosettes and are distributed across 4D-NT surface, magnified in D'. E) Quantitative analysis of mRNA levels detected by RT-qPCR of *CDH2* (N-cadherin) highlights neuralization levels in the 2D-uNT and 4D-NT samples. "Dev. Brain" represents a reference of a cDNA pool of developing human brain tissue. Data are represented as mean ± SD, with dots showing the mean of a technical triplicate and are shown as fold change in mRNA expression relative to *GAPDH*, a housekeeping gene, according to the $2^{-\Delta\Delta CT}$ method. $CTRL_2$ samples n=9; p-value > 0.05; One-way ANOVA. Scale bars: 500 µm in B and D, 100 µm in B', and 50 µm in B" and D'. Nuclei are counterstained with DAPI.

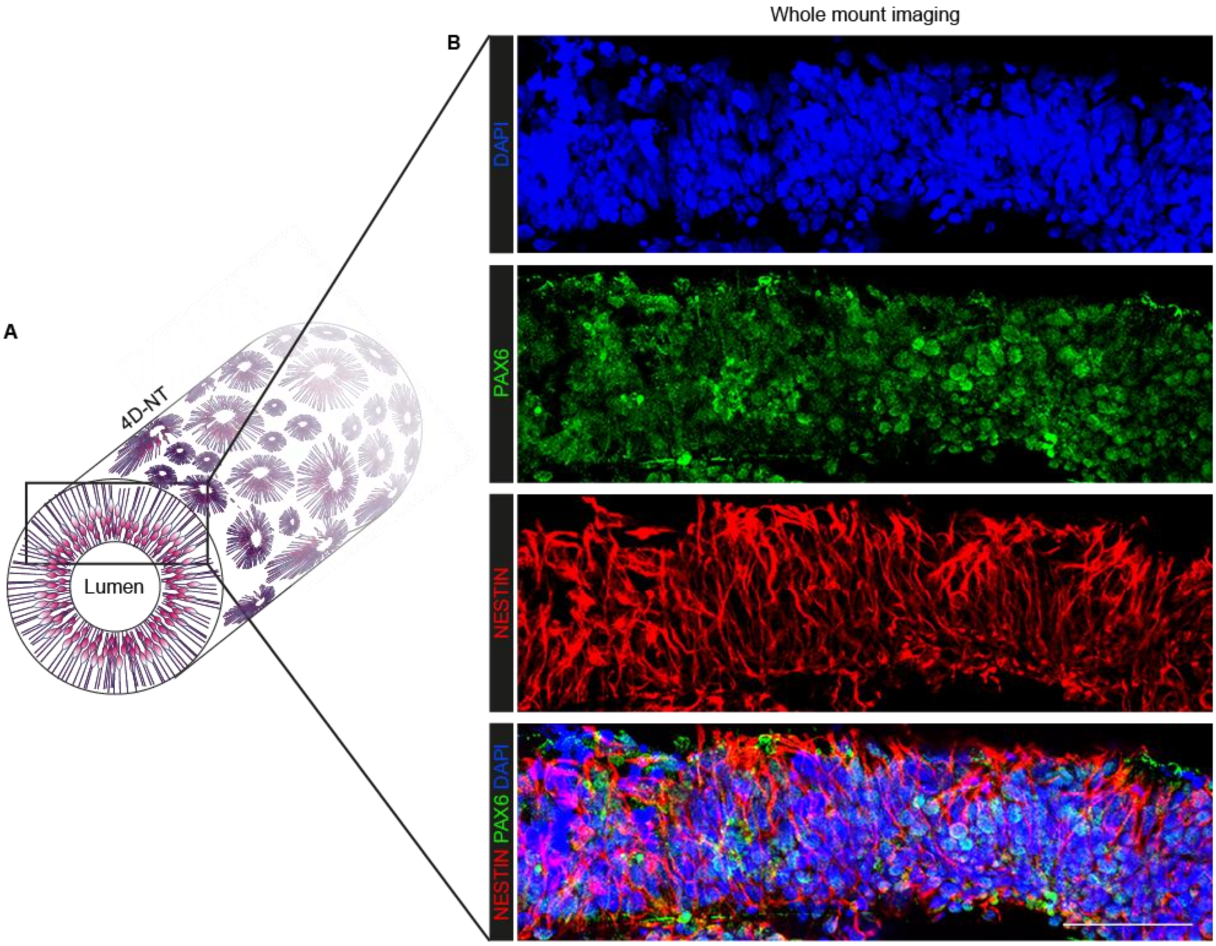

Figure S6: NPCs are radially oriented in 4D-NTs. A) Schematic representation of the spatial organization of the NPCs in 4D-NTs. B) Representative confocal images of 4D-NT showing PAX6$^+$ and nestin$^+$ NPCs radially oriented within the scaffold with respect to the ventricular ridge. Scale bar: 100 µm. Nuclei are counterstained with DAPI.

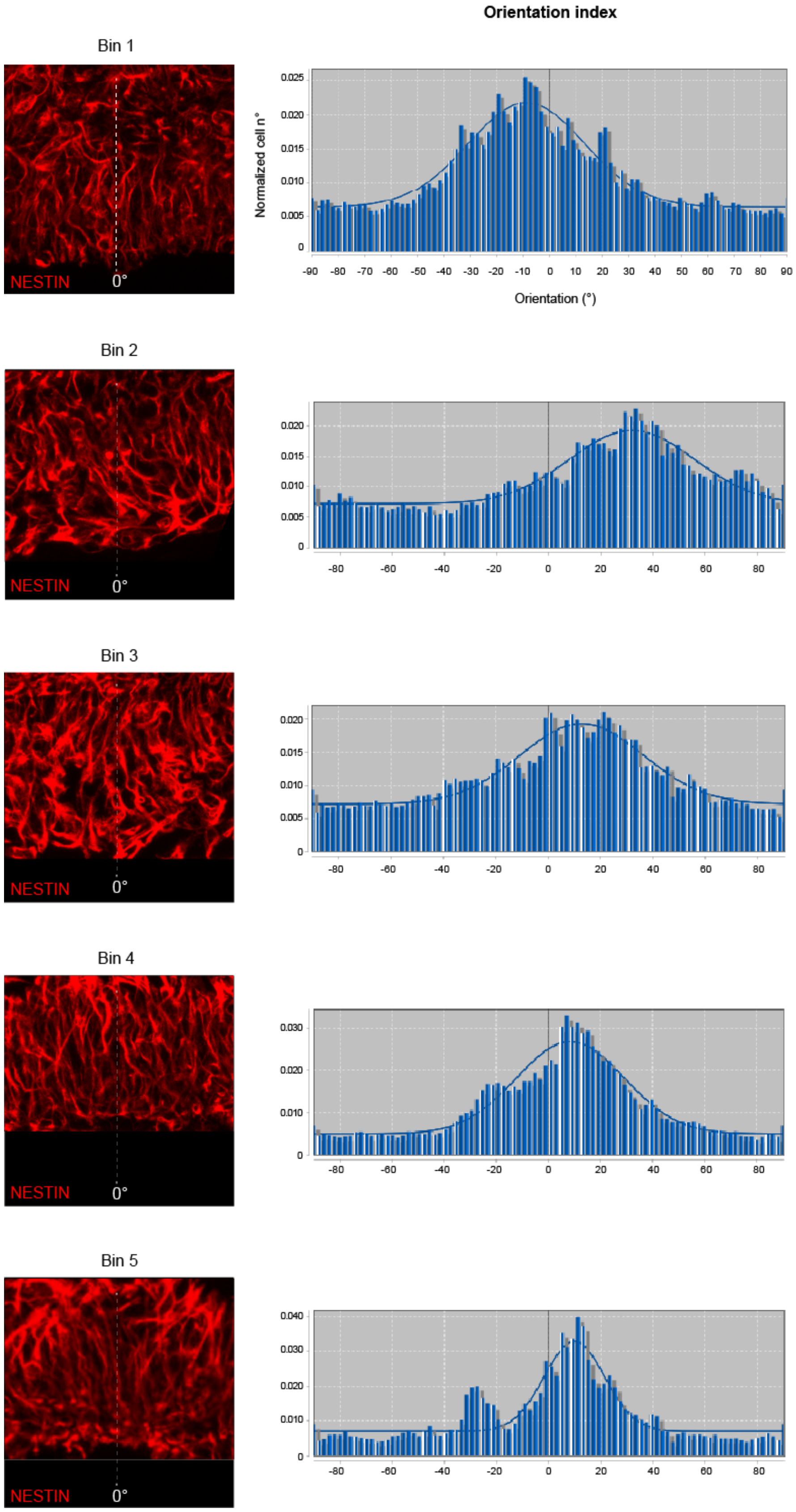

Figure S7: Orientation index analysis of nestin$^+$ processes in 4D-NTs: for each analyzed image (left), the histogram provided by the Directionality plug-in of ImageJ is shown.

Supplementary information:

Swelling tests

Swelling tests were performed on rectangular films (n = 10). Briefly, the dimensions of each sample were measured at dry state. Then, the films were placed in 1 ml PBS at 37 °C. Dimensions of each sample were evaluated after 24 h, and the swelling coefficient in each dimension was evaluated as

$$\boldsymbol{Swelling = \frac{L_f - L_0}{L_0} x\, 100}$$

where L_f is the dimension after 24 h, and L_0 is the dimension at dry state.

4D printing parameters

**Parameter**	**Value**
Print speed	5 mms^{-1}
Needle diameter	0.4 mm
Layer height	0.1 mm
Volumetric flow	0.4 mm^3s^{-1}

2D and 4D scaffold composition

The 2D non-folding and 4D folding scaffolds were designed using the same basic structure, consisting of a single layer made from GPTMS-GEL-15 bioink. The 2D scaffolds were fabricated by casting the GPTMS-GEL-15 layer. In contrast, 4D scaffolds were generated by printing GPTMS-GEL-5 stripes on top of the GPTMS-GEL-15 base layer on the internal side (Side I), resulting in a composite structure made of 70% GPTMS-GEL-15 and 30% GPTMS-GEL-5.
The surface percentages were measured by analyzing bright-field images of dry (non-folded) 4D scaffolds, specifically by calculating the surface area covered by GPTMS-GEL-5 stripes in relation to the total scaffold surface area. For this reason, since the majority of the surface area (70%) of the seeding side of the 4D scaffold is made by GPTMS-GEL-15, we used this formulation also for the 2D scaffold.

Cell morphology and behavior within the 4D-NTs, analyzed through wide-field and high-magnification confocal microscopy, showed no detectable changes in cellular orientation, morphology, or marker expression on the GPTMS-GEL-5 stripes in comparison with GPTMS-GEL-15.

Holder fabrication

To delay the scaffold folding, customized holders with an open central cavity (Figure 2B) were designed and fabricated in PDMS (Sylgard-184, Dow Corning, Michigan, USA) with a monomer/initiator ratio of 10:1 w/w, following the producer's protocol via indirect additive manufacturing.
